# The effect of maternal breast variations on neonatal weight gain in the first seven days of life

**DOI:** 10.1186/1746-4358-4-13

**Published:** 2009-11-18

**Authors:** Reza Vazirinejad, Shokoofeh Darakhshan, Abbas Esmaeili, Shiva Hadadian

**Affiliations:** 1 Social Medicine Department, Medical School, Rafsanjan University, Rafsanjan, Iran; 2 Medical School, Ali-Ebn Abitaleb Hospital, Rafsanjan University, Rafsanjan, Iran

## Abstract

**Background:**

This study aims to examine whether specific maternal breast variations (such as flat nipple, inverted nipple, large breast or/and large nipple) are barriers for weight gain in breastfed infants during the first seven days of life.

**Methods:**

In this prospective cohort study, 100 healthy term neonates were followed from birth to day seven in two groups; Group A: fifty neonates born to mothers with specified breast variations and Group B: fifty neonates born to mothers without such breast variations ("normal breasts"). All neonates were the first child of their families and there was no sex ratio difference between the two groups. Neonates' weight at birth and day seven were measured and the mean weight differences in the two groups were compared using paired t-test.

**Results:**

Neonates born to mothers without the specified breast variations had a mean weight gain of (+) 53 ± 154.4 g at day seven., Not only there was no increase in the mean weight of neonates in the other group, but they had a mean decrease of weight of (-) 162 ± 125.5 g by the seventh day of their life compared to birth weight. Thus, neonates born to mothers without breast variations had significantly greater weight gain than neonates born to the mothers with the specified variations (p < 0.01).

**Conclusion:**

Breast variation among first-time mothers acts as an important barrier to weight gain among breastfed neonates in the early days of life. Health professionals need skills in the management of breastfeeding among mothers with the specified breast variations, so that mothers are given appropriate advice on how to breastfeed and overcome these problems.

## Background

Breastfeeding success depends on appropriate attachment of the infant at the breast, in which the nipple and much of the areola are drawn well into the baby's mouth [1]. Anatomical variations of the breast, including flat nipple, inverted nipple, large breast and large nipple may act as barriers for the baby to latch on to the breast effectively. Babies need to have good attachment to the breast for successful breastfeeding and potential maternal problems such as these variations can make good attachment hard to achieve. Also, infant problems such as tongue-tie can be important [2-4].

Despite many studies conducted to explore factors associated with breastfeeding in both developed and developing communities [5-8], no investigation has been designed to show the effect of anatomical variations of the mother's breast on breastfeeding outcomes. Alexander et al considered inverted and non-protractile nipples as leading to problems establishing and maintaining breastfeeding [9]. They conducted a randomized controlled trial in Southampton, UK in 1987-1989, to determine the effect of two methods for resolving these problems among pregnant women with these breast variations who intended to breastfeed. In their study, "inverted nipple" was detected if "it was situated on a plane below the areola". They also reported that about 10% of pregnant women who intend to breastfeed have inverted or non-protractile nipples [9].

Few studies have been conducted to explore the association between "maternal obesity" and mothers' breastfeeding behavior and/or infants' weight gain. In Forster and colleagues' study, maternal obesity was negatively associated with breastfeeding outcomes [10]. Further, Mok et al conducted an analytical study and showed that infants born to obese breastfeeding mothers (Body Mass Index, BMI > 30 kg/m2) lost more weight in the first few days of their life and gained less weight in the first month compared to infants born to mothers with normal body mass index (18.5 kg/m2 < BMI < 25 kg/m2) [11]. Mothers with large breasts may experience similar problems to the mothers classified as obese in the Mok et al study [11].

This cohort study was designed to measure the effect of maternal breast variations, which may not be easily detected by first-time mothers themselves, on neonates' weight in the first week of life.

## Methods

This cohort study was conducted to evaluate the effect of breast variations in a group of first-time mothers on the weight gain of their infants in the first seven days of life. A cohort of 100 first-time mothers were recruited in the last few days of their pregnancy. They were referred to the Niknafs Maternity Hospital in Rafsanjan, Southeast Iran, between 1 February and 1 June 2006 for maternity care. This cohort was selected from approximately 1200-1600 pregnant women who were referred to the centre in this period.

In this four month period, 100 eligible mothers were consecutively invited to take part in the study. When a first-time pregnant woman with breast variation was recruited by the obstetrician to Group A, a first-time pregnant woman without this problem who was similar to her with respect to age (± 2 years), educational status, social class and living place was recruited to Group B. Fifty primiparous mothers and their healthy term neonates were selected into each group giving a total of 100 babies: Group A - 50 neonates born to mothers with at least one of the specified breast variations and Group B - 50 neonates born to mothers without any of the specified breast variations ("normal breasts"). Babies, who were not eligible, because they did not meet the inclusion criteria, were excluded from the study along with their mothers and were replaced with the next first-time mother who was eligible to participate.

Inclusion criteria were: mother aged between 18 and 30 years, natural vaginal birth, mother's education level between high school and diploma, desirable nutritional status of mother, annual family income equal or higher than moderate level of the annual salary of families living in the community, baby's birth weight more than 2500 g, mother intending to breastfeed her baby exclusively, being accessible from beginning to the end of data collection process, and no medical detectable barrier in babies or mothers to breastfeed. No mother used breast pumps or any other equipment to facilitate breast milk expression during the first seven days postpartum.

Data were collected on two occasions. At time 1, before giving birth, when mothers agreed to participate, maternal age, gestational age, social class, mother's educational status, living place, annual family income for assessing social class (Low = family annual salary less than 30,000,000 Rials, Moderate = family annual salary between 30,000,000 and 90,000,000 Rials, High = family annual salary more than 90,000,000 Rials) and the results of breast physical examination (by an obstetrician) were recorded. At time 2, after the birth, data on neonate's gender, health status at birth, birth weight (using a standard scale), neonate's health status on day seven and weight on day seven of life (using the standard scale) were collected.

In order to reduce bias in this study, not only were the participants not informed about the presence or absence of the abnormality, but the obstetrician and statistician were also not aware of the babies' weight or group allocation.

**Figure 1:**
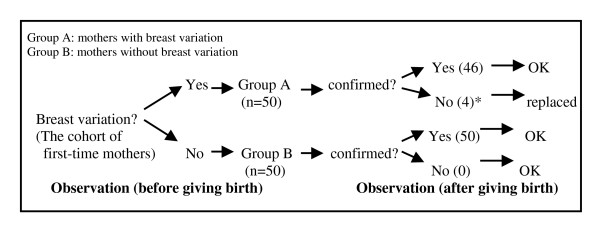
**Recruiting process of participants**. *Four mothers diagnosed as having "large breasts" in pregnancy, were found to have "normal breasts" after birth, and were replaced with four mothers with confirmed breast variation.

Variations were detected on physical examination if there was any form of "large nipple", "flat nipple", "inverted nipple" and "abnormally large breast". In this study; a nipple was defined as abnormally large, abnormally flat or inverted, or the breast was defined as abnormally large, if it was impossible for the first-time mother to breastfeed her baby normally without receiving help from others and/or equipment. These variations were confirmed by the obstetrician once she observed the breastfeeding process after the birth. Since mothers were assigned to the two groups before giving birth, it was possible for the obstetrician to have made a mistake in group allocation. Therefore, if the obstetrician realized that her diagnosis was not correct after watching the breastfeeding process, the mother and her baby were excluded or allocated to the other group. The process of recruiting participants in the two groups of mothers with and without breast variation is presented in Figure 1. There were four mothers diagnosed as having large breasts "before giving birth" whose were not confirmed in the stage "after giving birth" and were replaced with four mothers with confirmed breast variation.

A pilot study was conducted on twenty mothers and their babies in two groups of equal size (ten in each group) for the purpose of sample size determination. Estimating an effect size of 0.6 with a 95% confidence interval (α = 0.05) and 80% power (β = 0.2) [12], a sample size of 45 subjects for each group was required. Considering about 10% dropout of respondents, 50 eligible mothers and their neonates were selected for each group.

Data were analyzed using parametric (paired t-test) and non-parametric (chi-Square, Mann-Whitney U) tests where appropriate, using SPSS (Version 12) for Windows. For each neonate, the difference in weight between the seventh day of life and birthweight on the first day of life was calculated. Then, for each group, a mean difference of weight was calculated by dividing the sum of the differences by 50 (the number of neonates in each group). The difference of each neonate's weight at birth and day seven was calculated and the mean weight differences in the two groups were compared using paired t-test. The mean weights and mean weight differences are presented as mean ± standard deviation (SD).

For the purpose of data collection, ethical approval was obtained from Rafsanjan University of Medical Sciences. All participants were given an information sheet about the study and gave written consent.

## Results

The cohort consisted of 100 primiparous mothers and their neonates, 50 pairs in each group. Only one mother declined to participate and was replaced with another eligible mother. Some participant characteristics are presented in Table 1. The groups were similar with respect to maternal age, education, family income (social class), infant sex and gestational age.

**Table 1:** Demographic characteristics of the participants in the two groups and the type of breast variation

Variables	Groups			
	
	Group A With specified variations (n = 50)		Group B Normal breast (n = 50)	
	n	%	n	%
Maternal age (years)**				
<20	7	14	7	14
20-29	31	62	30	60
30+	12	24	13	26

Maternal education*				
did not complete high school	18	36	19	38
completed high school	25	50	23	46
Higher	7	14	8	16

Annual family income**				
Low	0	0	1	2
Moderate	39	78	41	82
High	11	22	8	16

Breast variation***				
flat nipple	27	54	0	0
large nipple	17	34	0	0
large breast	12	24	0	0
inverted nipple	7	14	0	0

Infant birth weight (g)				
< 3000	16	32	14	28
3000-3999	32	64	34	68
≥4000	2	4	2	4

Infant gender*				
Male	21	24	20	40
Female	29	58	30	60

Gestational age (weeks)*				
37-38	10	20	9	18
39-40	28	56	29	58
41-42	12	24	12	24

*Chi-Square showed no significant difference between the two groups.

**t-test and Mann-Whitney U test showed no significant difference between the two groups.

***About one-quarter of mothers with breast variations (n = 13) had more than one variation.

The weights of neonates born to the two groups of mothers at birth and on day seven of the baby's life are presented in Table 2. There was no significant difference in birth weight between the two groups of neonates: mean birth weight of neonates born to mothers with and without breast variations was 3246 ± 480 g and 3185 ± 450 g, respectively. The mean weight of neonates born to mothers with and without breast variations on the seventh day of life was 3084 ± 427 g and 3238 ± 490 g, respectively.

**Table 2:** Comparison of the weight of neonates born to mothers with and without specified breast variations at birth and on day seven

	Timing		
		
Groups	At birth	Day 7	Mean weight gain (+)/loss (-) (g)
**A: Abnormal breast (n = 50)**	3246 ± 480	3084 ± 427	-162 ± 125.5

**B: Normal breast (n = 50)**	3238 ± 490	3185 ± 450	+53 ± 154.4

Paired t-test	p = 0.515	p = 0.09	p = 0.00

The mean difference in neonates' weight between birth and day seven, among neonates who were born to mothers without breast variations (53 ± 154.4 g) was significantly larger than the mean difference of neonates' weight in the other group (-162 ± 125.5 g) (Table 2). The values of differences were normally distributed (one-sample Kolmogorov-Smirnov test). Paired t-test used for comparing these two means of differences showed significant difference (t = 7.5, df = 49, p < 0.01).

## Discussion

There are a number of limitations in this study, such as the absence of breast/nipple measurements and scientific definitions for breast variations, and the lack of data on infant outcome according to the type of breast variation, as well as follow-up on duration of breastfeeding. However, the results showed that the mothers in the two groups were successfully matched based on maternal age, maternal education, annual family income and gestational age and infant birth weight. Therefore, the effect of these factors on the weight of neonates was eliminated and this increased the specificity and accuracy of the association obtained in this investigation.

We found that the mean weight of neonates who were born to mothers with at least one type of breast variation was less than their birth weight on the seventh day of life. Whereas, the mean weight of neonates who were born to mothers without these variations, was over birth weight and significantly higher than the weight of infants of the mothers with breast variations. Therefore, the breast variations identified in this study, and possibly other variations as well, could cause breastfeeding and consequent weight gain problems that can be seen as early as day seven of life. Without clinical breastfeeding intervention, these variations can lead to the addition of infant formula which can have profoundly negative health consequences.

Other studies have attempted to discover factors affecting breastfeeding behaviour [13,14], as well as investigations designed to increase breastfeeding prevalence [15]. Many studies have shown an association between maternal obesity and breastfeeding behavior [11,16]. However, to our knowledge, no previous investigation of the effect of maternal breast variations on infant weight gain has been reported. McAllen wrote that not only are large breasts not barriers for breastfeeding but they can also have benefits if mothers know how to breastfeed their babies [17]. Therefore, if, in particular, first-time mothers are aware of their breast variation and know how to overcome the problem, breast variations need not have a negative effect on their breastfeeding and neonatal weight gain. The results of our study showed that breast variations can play an important role in weight gain among neonates born to first-time mothers. In return, this problem can also affect first-time mothers' intention to breastfeed and persuade them to change their feeding behavior in future. It has been shown that previous breastfeeding experience is one of the predictive factors of breastfeeding intention among mothers [5,11]. However, due to the need in our study to assist mothers who were identified as experiencing difficulties, using interventions such as bottle feeding (of expressed breast milk) the effect of breast variations on weight gain among neonates was only explored in the first few days of their life.

The other point that makes our findings important is the speed of neonates' growth in the first few weeks of their life, as well as missing the chance of receiving benefits from colostrum which is produced in the early days of neonates' life [18,19]. The speed of neonates' growth is rapid in the months after birth, and then slows. It is reported that the most rapid period of growth is in the early days of babies' life [20].

Although further studies are needed to confirm these findings, our results suggest that all first-time mothers should be physically examined by maternity care staff and necessary intervention or/and proper advice should be given to those who suffer from at least one type of breast variation. This can be a part of routine examination that pregnant women receive. Randomised controlled trials are needed to evaluate methods which are being used to overcome this problem and/or to present new methods. More attention should be paid to this problem, in particular, in communities with a high prevalence of maternal breast variations.

## Conclusion

Our findings showed that the effect of mothers' breast variations on neonates' weight gain can be considered important enough to provide routine examination of pregnant mothers' breasts. This is not a routine program in all communities worldwide. However, more investigations are strongly recommended. Based on our results, health professionals need to develop skills in the management of breastfeeding among mothers with these problems, so that mothers are given appropriate advice on how to counteract breastfeeding difficulties. Further research is needed to explore long-term effects of maternal breast/nipple variations on the health of their infants.

## Competing interests

The authors declare that they have no competing interests.

## Authors' contributions

RV had primary responsibility for designing the study, evaluation and preparation of this manuscript and data analysis. SD participated and supervised data collection process and method development. AE helped with data analysis and evaluation of this manuscript. SH contributed to the study design and data collection.
